# Establishment of a Real-Time PCR Assay for the Detection of *Devriesea agamarum* in Lizards

**DOI:** 10.3390/ani13050881

**Published:** 2023-02-28

**Authors:** Maria Brockmann, Christoph Leineweber, Tom Hellebuyck, An Martel, Frank Pasmans, Michaela Gentil, Elisabeth Müller, Rachel E. Marschang

**Affiliations:** 1 Laboklin GmbH & Co. KG, Steubenstr. 4, 97688 Bad Kissingen, Germany; 2 Department of Pathobiology, Pharmacology and Zoological Medicine, Ghent University, Salisburylaan 133, B-9820 Merelbeke, Belgium

**Keywords:** *Devriesea agamarum*, polymerase chain reaction, *Uromastyx* sp., *Pogona vitticeps*, bearded dragon, lizard, reptile, cheilitis, dermatitis

## Abstract

**Simple Summary:**

Bacterial infections can play an important role in dermatitis in lizards. The bacterial species *Devriesea* (*D*.) *agamarum* is a known cause of dermatitis, cheilitis and even fatal disease in lizards. Disease has most often been reported in *Uromastyx* species, but other lizards may also be affected. However, some are asymptomatic carriers, increasing the risk of spreading *D. agamarum*. Usually, *D. agamarum* is detected with culture-based methods. It was the aim of this study to establish a real-time PCR assay to expand diagnostic options in routine diagnostics. The presented assay is able to detect *D. agamarum* in clinical samples, decreasing laboratory turn-around time in comparison to conventional culture-based detection methods. This enables a fast therapeutic approach for affected animals and decreases the risk of spread.

**Abstract:**

(1) Background: *Devriesea* (*D.*) *agamarum* is a potential cause of dermatitis and cheilitis in lizards. The aim of this study was to establish a real-time PCR assay for the detection of *D. agamarum*. (2) Methods: Primers and probe were selected targeting the 16S rRNA gene, using sequences of 16S rRNA genes of *D. agamarum* as well as of other bacterial species derived from GenBank. The PCR assay was tested with 14 positive controls of different *D. agamarum* cultures as well as with 34 negative controls of various non-*D. agamarum* bacterial cultures. Additionally, samples of 38 lizards, mostly *Uromastyx* spp. and *Pogona* spp., submitted to a commercial veterinary laboratory were tested for the presence of *D. agamarum* using the established protocol. (3) Results: Concentrations of as low as 2 × 10^4^ colonies per mL were detectable using dilutions of bacterial cell culture (corresponding to approximately 200 CFU per PCR). The assay resulted in an intraassay percent of coefficient of variation (CV) of 1.31% and an interassay CV of 1.80%. (4) Conclusions: The presented assay is able to detect *D. agamarum* in clinical samples, decreasing laboratory turn-around time in comparison to conventional culture-based detection methods.

## 1. Introduction

*Devriesea* (*D*.) *agamarum* is a bacterial species known to cause dermatitis and cheilitis in lizards. Disease has most often been described in *Uromastyx* spp. [1,2,3,4,5]. However, *D. agamarum* can also infect other lizards [6,7]. It has been reported in captive [8,9] as well as in free-ranging lizards [7]. Clinical signs of disease generally include dermatitis or cheilitis, often described with a yellow crusty appearance [10]. Disease outbreaks with extensive mortality have also been reported, especially if lizards developed septicaemia. [7,11]. Bearded dragons have been described to asymptomatically carry *D. agamarum* in their oral cavities [2,3]. Treatment of affected animals usually includes debridement of dermal lesions and systemic use of antibiotics—especially cephalosporines are considered effective [4,12]—and may also require disinfection of the enclosure [13]. Autovaccines have also been discussed as a treatment method [14].

Therefore, a fast and reliable diagnostic approach is an important consideration, both in clinical disease with suspected *D. agamarum* infection and in entry controls. *D. agamarum* is relatively easily cultured at 37 °C but also grows at temperatures of 25−42 °C on Columbia agar with 5% sheep blood [11]. Diagnosis can, however, be complicated in laboratories with limited experience with this pathogen. Matrix-Assisted Laser Desorption/Ionisation Time-of-Flight Mass Spectrometry (MALDI-TOF MS), one of the most frequently used standard techniques for the identification of bacteria in routine laboratory diagnostics, may not (yet) be able to identify *D. agamarum* when working with standardized databases [15]. However, this issue is likely to be overcome as databases expand. Currently, laboratories can improve *D. agamarum* identification by implementing and expanding their own MALDI-TOF MS databases or using 16S rRNA gene sequencing to identify cultured but unidentified isolates. Another option would be a specific PCR assay for *D. agamarum,* which might prove especially valuable if other infectious agents, such as viral or fungal pathogens, are also suspected, allowing concurrent testing from the same sample. The aim of this study was, therefore, to develop a PCR assay for the detection of *D. agamarum*.

## 2. Materials and Methods

### 2.1. Bacterial Isolates Used to Establish the qPCR

In total, 14 *D. agamarum* isolates were used in this study as positive controls.

Three *D. agamarum* isolates (GenBank accession numbers: MT664091-93) were obtained from routine diagnostic submissions at Laboklin GmbH & Co. KG (Bad Kissingen Germany) in 2019 [15], while 11 were isolated between 2005 and 2009 at the Faculty of Veterinary Medicine, Ghent University (Table 1). Non-*D. agamarum* isolates (n = 34) were obtained from the German Collection of Microorganisms and Cell Cultures (DSMZ, Braunschweig, Germany). Some were included in order to determine the ability of the assay to exclude a broad spectrum of different bacterial species. Others, like *Brachybacterium* sp. or *Dermabacter* sp., were included as their sequences were described to be highly similar to *D. agamarum* [11] (Table 2). These 34 isolates served to determine the specificity of the PCR.

### 2.2. DNA Preparation

Pure cultures of each strain were incubated in 750 μL lysis buffer (MagNA Pure DNA Tissue Lysis Buffer, Roche, Mannheim, Germany) and 75 μL proteinase K (proteinase K, lyophilisiert, ≥30 U/mg, Carl Roth GmbH & Co KG, Karlsruhe, Germany) for one hour at 65 °C. From this, 200 μL were utilized for automated nucleic acid (NA) extraction using the MagNA Pure 96 DNA and Viral NA Small Volume Kit (Roche, Mannheim, Germany) according to the manufacturer’s instructions. The resulting NAs were eluted in a volume of 100 μL. The isolated NAs were kept at −18 °C until the PCR tests were performed. The DNA used for the dilution series was extracted manually using the QIAamp^®^ DNA MicroKit (50) (Qiagen, Hilden, Germany), and the DNA concentration was measured with a spectrophotometer (NanoDrop 2000, Thermo Fisher Scientific, Inc., Wilmington, NC, USA).

### 2.3. Design of Primers and Oligonucleotide Probe, PCR Protocol and Optimisation of Annealing Temperature

The 16S rRNA gene was selected as the target region based on the availability of sequence data from a variety of isolates. Sequences of *D. agamarum* (NZ_LN849456.1, LN849456.1, NR_044368.1, EU009865.1, KF647330.1) were retrieved from GenBank, and multiple sequence alignment was performed with other sequences of different bacterial species (e.g., *Agromyces* species, *Arthobacter* species, *Brachybacterium* species, *Dermabacter* species, *Pseudomonas* species) using MUSCLE (https://www.ebi.ac.uk/Tools/msa/muscle/ last accessed on 20 February 2023). The primers and probe were designed using primer3 (https://primer3.ut.ee/ last accessed on 20 February 2023).

Reactions included 1.0 µL of each primer (10 µM), 1.0 µL of the probe (2 µM), 4.0 µL DNA Process Control Detection Kit qPCR Reaction Mix and 5.0 µL template DNA in a total volume of 20.0 µL. Amplification was performed with a LightCycler 96 (Roche, Mannheim, Germany) in a 96-well format.

**Figure 1:**
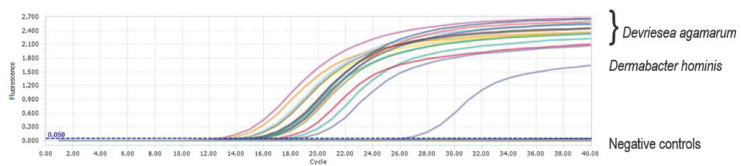
Two-step amplification carried out at 60 °C: All *Devriesea agamarum* isolates tested resulted in positive signals with CT values ranging from 12.61 to 18.54. *Dermabacter hominis* (blue curve) also gave a positive signal with a CT value of 26.29.

The following protocol was used: Preincubation at 95 °C for 30 s followed by 40 cycles of two-step-amplification (95 °C for 5 s and 60 °C for 30 s). PCR-grade water (Roche, Mannheim, Germany) served as a negative control. While all other bacterial samples were negative, *Dermabacter hominis* produced positive PCR results at an annealing temperature of 60 °C (Figure 1). Therefore, DNA of *Dermabacter hominis* (DSM 30958) from the DSMZ as well as DNA of *D. agamarum* (GenBank Accession number: MT664092.1/0919Ur) [15], was tested in duplicate with different annealing temperatures (protocol 1: 65.0 °C, protocol 2: 66.0 °C, protocol 3: 67.0 °C) using a LightCycler 96 (Roche, Mannheim, Germany).

### 2.4. Determination of Specificity at 66 °C and of Repeatability and Sensitivity of the Assay

Various non-*D. agamarum* bacterial species (Table 2) were tested in duplicate for positive reactions at 66 °C. The following protocol was used: Preincubation at 95 °C for 30 s followed by 40 cycles of two-step-amplification (95 °C for 5 s and 66 °C for 30 s). PCR-grade water (Roche, Mannheim, Germany) served as a negative control. DNA of 14 *D. agamarum* isolates (Table 1) was also tested.

To assess the intraassay repeatability of the PCR, standard deviations were calculated for 10-fold serial dilutions in triplicate on a single plate. For the interassay reproducibility, standard deviations were calculated for a 10-fold serial dilution series which was amplified three times daily for two days. The standard deviations of the CT values were used to calculate the percent of coefficient of variation (CV%).

Detection limit for bacterial cell dilutions: In order to determine the sensitivity of the assay, a culture of *D. agamarum* (isolate MT664091.1/0219Bf) was used, starting with a dilution (D0) of 0.5 McFarland (1.5 × 10^8^ per mL). This suspension (D0) was 10-fold serially diluted (D1–D10) in duplicate, and 1 mL of each dilution was inoculated onto Columbia agar with defibrinated sheep blood (Becton Dickinson GmbH, Heidelberg, Germany/Oxoid GmbH, Wesel, Germany), incubated at 36 °C and checked for growth after 30 h. Colony forming units (CFU) were counted if the result was expected to be between 0 and 300 CFU. These results were then used to determine the limit of detection of the assay. DNA was extracted from 200 µL of each dilution (D1–D10) as described above, and PCR was carried out in duplicate. The detection of *Dermabacter hominis* (DSM 30958) was quantified in the same way.

Detection limit for DNA from pure culture: To evaluate the assay’s sensitivity, PCR was carried out in triplicate using serial 10-fold dilutions of DNA prepared from colonies of *D. agamarum* (isolate MT664091.1/0219Bf).

Spiked-in matrix: The assay was also evaluated using a spiked-in matrix (*D. agamarum*-negative-tested lizard skin with a known concentration of target DNA). For these assays, 10 µL of the above-described dilutions D0 to D6 were inoculated onto *D. agamarum*-negative-tested lizard skin. The skin was incubated in 500 µL lysis buffer, and 50 µL proteinase K and NA were extracted from 200 µL of this suspension as described above and eluted in a total volume of 100 µL NA. The PCR was carried out in duplicate as described above.

### 2.5. Testing of Samples Submitted to a Commercial Veterinary Laboratory

Clinical samples from lizards for which appropriate material (skin, crusts, dry swab) was submitted to a commercial veterinary laboratory between March 2022 and December 2022 and for which the submitting veterinarian indicated an interest in *D. agamarum* diagnostics were tested using the established protocol to evaluate the PCR for use with clinical samples. Some of the samples were derived from animals showing clinical signs, others from asymptomatic animals tested in the context of a health check—often when *D. agamarum* had been isolated from animals in the group previously. If suitable material was available (skin or swab in a transport medium), bacteriology was performed (as previously described [15]). Identification of isolates was based on growth characteristics on agar plates (Columbia Agar with defibrinated sheep blood and Endo Agar, Becton Dickinson GmbH, Heidelberg, Germany), biochemical parameters and MALDI-TOF MS. In doubt, colonies were also re-checked via PCR. If the results of the bacteriological culture were available, the results of the PCR and culture would be compared. Samples were considered PCR positive if the cycle threshold (CT) was <35.0 and equivocal if the CT was ≥35.0, but a signal was obtained. If swabs of different origins regarding the localisation (e.g., dermal and oral) of the same animal were received, all samples were tested separately. Amplicons from positive samples were sequenced (ABI PRISM 3130 XL Genetic Analyser, Applied Biosystems, Foster City, CA, USA) and sequences were analysed by BLAST (https://blast.ncbi.nlm.nih.gov/Blast.cgi, last accessed on 20 February 2023).

## 3. Results

### 3.1. Development of the PCR

The selected primer and probe sequences (Eurofins MWG Operon, Ebersberg, Germany) are shown in Table 3. The product size was expected to be 246 base pairs.

Optimisation of annealing temperature was performed using three protocols with different annealing temperatures. In protocol 1 (65 °C), *D. agamarum* DNA was detected with CT values of 14.58 and 16.49 and *Dermabacter hominis* with CT values of 29.43 and 29.62. In protocol 2 (66 °C), *D. agamarum* DNA was detected with CT values of 16.22 and 16.14, and *Dermabacter hominis* showed values of 34.28 and 35.19. In protocol 3 (67 °C), *D. agamarum* was detected with CT values of 15.57 and 16.57, while *Dermabacter hominis* was not detected. Therefore, an annealing temperature of 66 °C was chosen for all further analyses as it was considered sufficient to discriminate between pure cultures of *D. agamarum* and *Dermabacter hominis* DNA without losing sensitivity for the detection of *D. agamarum*.

### 3.2. Specificity, Repeatability and Sensitivity of the Assay

Specificity of the assay using the protocol with 66 °C as an annealing temperature was determined using 14 *D. agamarum* isolates (Table 1) and 34 non-*D. agamarum* bacterial isolates (Table 2) in duplicate.

No signal was obtained from any bacterial DNA from isolates other than *D. agamarum* except for *Dermabacter hominis*. The CT value obtained using the DNA from pure cultures of *Dermabacter hominis* were high (34.55 and 34.48) in comparison to those reached using DNA from *D. agamarum* (12.08–18.14) but still below the threshold set for clinical samples. As these are the results for DNA extracted from pure culture, 66 °C was considered sufficient for further testing of samples without prior cultivation as samples without prior cultivation are expected to yield a lower pathogen level. The intraassay CV was calculated to be 1.31%, while the interassay CV was 1.80%.

Detection limit for bacterial cell dilutions: A positive PCR signal was detected for *D. agamarum* dilutions D0–D4. An equivocal signal was detected for D5 and D6. In culture, D4 corresponded to 2 × 10^4^ colonies per mL. Therefore, the assay sensitivity was 2 × 10^4^ colonies per mL with dilutions of bacterial cell culture serving as a template. This corresponds to approximately 200 CFU per PCR. For the serially diluted culture of *Dermabacter hominis,* no positive PCR signals were observed. Two dilutions (D0 and D1) resulted in equivocal CT values, with D0 being set to 0.5 McFarland (1.5 × 10^8^ per mL).

Detection limit for DNA from pure culture: The DNA concentration was determined to be 42.5 ng/µL (A260/A280: 1.94). *D. agamarum* DNA was detectable in dilutions up to 1:10^5^. Therefore, DNA concentrations of as low as 425 fg/µL were detectable in the PCR.

Spiked-in matrix: Spiked-in matrixes produced clearly positive results up to skin spiked with 10 µL of D2 with D2 corresponding to 2 × 10^6^ colonies per mL (approximately 360 CFU per PCR considering dilution during sample preparation).

### 3.3. Testing of Clinical Samples

In order to test the use of the developed PCR for clinical samples, a total of 48 samples from 38 lizards were tested for the presence of *D. agamarum* (Table 4). The samples were derived from several species, mostly agamids (*Pogona* spp. and *Uromastyx* spp.), and were of different origins (zoological collection, animal rescue centre, private owner) from Germany and the Netherlands. Some of these animals were asymptomatic and were tested in the context of a health check. Others showed clinical signs such as skin lesions, hyperkeratosis or stomatitis (Table 4). Samples were mostly derived from the oral cavity or skin/crusts. Of the 38 animals tested, *D. agamarum* was detected by PCR in 16 animals (42.10%). A further five animals (13.16%) were considered to have equivocal results, and 17 animals (44.74%) were negative for *D. agamarum*. One animal (animal 3) that tested negative proved to be infected with a fungus of the family Onygenaceae. A bacteriological examination was performed for 33 of the 38 animals, but *D. agamarum* was not cultured. However, in most of these cases, various other bacterial species (Table 4) were cultured when bacteriology was performed.

## 4. Discussion

*D. agamarum* is an important pathogen causing skin lesions and, in some cases, systemic disease in lizards. Depending on the species, some animals can be inapparent carriers, while others may develop severe diseases. Diagnosis of the causative agent is therefore important in order to facilitate treatment as well as to prevent the spread of disease. Since animals may suffer when untreated and the risks of spreading increase with time, a fast diagnostic approach is important. The detection of *D. agamarum* is commonly achieved via culture, followed in some cases by 16S rRNA gene sequencing [11,16].

The PCR developed in this study provides a time-saving tool compared to culture and bacterial identification. Detection of *D. agamarum* and concurrent bacteriological examination was performed in 33 of the 38 animals, resulting in 13 of 33 clearly PCR-positive animals but no culture-positives. *D. agamarum* is expected to be abundantly present in symptomatic animals. Culturing of *D. agamarum* is not considered difficult and has been successfully performed in this laboratory before [15]. However, a successful culture depends on the quality of the submitted samples. Appropriate samples include affected tissue below hyperkeratotic crusts or inside of the crusts as well as organs in septicaemic lizards and subcutaneous granulomas. In asymptomatic animals as well as in symptomatic animals, isolation from the oral cavity, gastrointestinal tract or healthy skin may be challenging. *D. agamarum* was cultured in the laboratory in which the study was performed during the study period, but these samples were excluded from the study as no suitable material for concurrent PCR testing (e.g., dry swab, skin) was available. In this study, six animals (1, 2, 21 and 22–24) were known to have been symptomatic and had positive PCR results. Bacteriological culture was performed in three of these animals (22–24). In animal 24, a positive PCR result was only obtained from the skin sample, which was not tested by bacteriological culture. Animal 6 might have been symptomatic (no information was received, but due to the reported previous treatment, it seemed likely). It was treated with antibiotics prior to sampling, which might have influenced the bacteriology results. Possible reasons for the failure of culturing *D. agamarum* out of positive clinical samples in this study include previous antibiotic treatment, incorrect sampling techniques, contamination with (oral) microbiota, increased transport time, inappropriate transport conditions or overgrowth by other bacteria. The latter is especially important as in the presented cases, no selective media for gram-positive bacteria were used, and various different bacterial species are expected to be present on the skin [17,18].

PCR analysis is useful if the performance of bacterial culture is difficult, e.g., due to previous treatment with antibiotics, inadequate preanalytical conditions (such as increased or decreased temperature, increased transport time, inadequate transport medium), or in cases in which overgrowth by other bacteria make detection challenging or impossible. The detection of *D. agamarum* via PCR can also simplify concurrent PCR testing for other known pathogens, e.g., viral or fungal pathogens known to cause dermatitis [19,20], since the same extracted nucleic acids can be used. In general, PCR is advantageous when culturable samples are unavailable, for example, when older samples are tested or stored DNA is examined. However, bacterial DNA can persist in the environment [21], and *D. agamarum* has been shown to survive for several months in the environment, depending on the conditions [13]. A PCR could therefore detect bacteria even in cases in which these were not responsible for clinical signs or in which no replication-competent bacteria were present.

The PCR developed here was not 100% specific. Pure cultures of *Dermabacter hominis* did result in a weak positive signal. However, if diluted, only equivocal results were observed. *Dermabacter hominis* is genetically closely related to *D. agamarum* [2,11,22]. *Dermabacter hominis* is associated with the human microbiome [23]. It is occasionally described in human clinical samples such as abscesses or blood cultures [24,25,26] but is usually found to be of minor clinical significance [27]. So far, its clinical importance in reptiles is very unclear. Contamination during sampling or sample preparation should be considered a possible option leading to false equivocal results. However, clinical samples are expected to yield less bacterial DNA, making false equivocal results less likely. The 16S rRNA gene is known to be highly conserved between bacterial species, which makes it a useful target if the aim is to identify different bacterial species. It is a commonly used target for bacterial detection, and therefore a large amount of sequence data is available for a wide range of bacterial species. However, it may not be ideal for differentiating closely related bacteria. Currently, the availability of sequence data for *D. agamarum* other than the 16S rRNA gene is limited, but in the future, other targets may prove to be better options. In the meantime, especially equivocal CT values should be evaluated with caution in the face of clinical signs, sampling and sample preparation, and ideally, retesting is recommended. Possibly, skin samples might prove more useful than swabs as they yielded lower CT values in two of the three animals for which both sample types were available, but this might be highly dependent on the sampling method.

The PCR protocol developed in this study proved helpful for the detection of *D. agamarum* in clinical samples. *D. agamarum* was detected in oral swabs from clinically healthy *Pogona* species and serrated casquehead iguana (*Laemanctus serratus*), while the results in which equivocal results were obtained were also from clinically healthy *Pogona* species as well as from clinically healthy black hardun (*Laudakia stellio picea)*. *Pogona* species have previously been shown to be possible inapparent carriers of *D. agamarum* and a possible source of infection for more sensitive species [2,3].

Therefore, this PCR protocol may not only be useful for clinical cases but also as a screening tool. However, the number of tested samples is still small, and testing of larger sample numbers is necessary in order to confirm the usefulness of this method for clinical practice.

## 5. Conclusions

A real-time PCR was developed that is able to detect *D. agamarum* in clinical samples. The assay provides a fast method for the detection of this important pathogen of lizards but should be evaluated with further samples in the clinical context.

## Figures and Tables

**Table 1:** *Devriesea agamarum* isolates used to establish the PCR.

No.	Isolate	Host	Sample Material	Laboratory
1	MT664091.1/0219 Bf	*Brachylophus fasciatus*	Swab (skin)	Laboklin GmbH & Co. KG, Germany
2	MT664092.1/0919 Ur	*Uromastyx* sp.	Swab (skin)	Laboklin GmbH & Co. KG, Germany
3	MT664093.1/0319 Ur	*Uromastyx* sp.	Swab (skin)	Laboklin GmbH & Co. KG, Germany
4	IMP2 vacc	*Agama impalearis*	Swab (dermatitis), liver	Faculty of Veterinary Medicine, Ghent University
5	30.7	*Uromastyx dispar*	Swab (dermatitis, cheilitis)	Faculty of Veterinary Medicine, Ghent University
6	34.1	*Uromastyx acanthinura*	Swab (dermal abscess)	Faculty of Veterinary Medicine, Ghent University
7	23	*Pogona vitticeps*	Swab (oral cavity)	Faculty of Veterinary Medicine, Ghent University
8	24	*Pogona vitticeps*	Swab (oral cavity)	Faculty of Veterinary Medicine, Ghent University
9	25	*Pogona vitticeps*	Swab (oral cavity)	Faculty of Veterinary Medicine, Ghent University
10	26	*Crotaphytus collaris*	Swab (dermatitis)	Faculty of Veterinary Medicine, Ghent University
11	28	*Eublepharis macularius*	Swab (oral cavity)	Faculty of Veterinary Medicine, Ghent University
12	30b	*Ctenonotus gingivus*	Swab (cloaca)	Faculty of Veterinary Medicine, Ghent University
13	4d	*Iguana delicatissima*	Swab (dermal abscess)	Faculty of Veterinary Medicine, Ghent University
14	L26	*Pogona vitticeps*	Swab (oral cavity)	Faculty of Veterinary Medicine, Ghent University

**Table 2:** Bacterial species used as negative controls to determine the specificity of the PCR.

No.	Bacterial Species	DSMZ Number	Original Depositor (Acc. to DSMZ)
1	*Acinetobacter baumannii*	DSM 30007	J.V. Cook
2	*Bacillus atrophaeus*	DSM 675	E. McCoy
3	*Cytobacillus firmus*	DSM 359	G. Bredemann
4	*Brachybacterium faecium*	DSM 4810	H.E. Schefferle
5	*Dermabacter hominis*	DSM 30958	C. Moissl-Eichinger
6	*Enterococcus faecalis*	DSM 2570	Kaiser-Permanente
7	*Enterococcus faecium*	DSM 20477	A. Grumbach
8	*Enterococcus faecium*	DSM 2146	J.M. Skerman (*Streptococcus faecalis)*
9	*Escherichia coli*	DSM 1103	F. Schoenknecht
10	*Escherichia coli*	DSM 1576	G.C. Crooks
11	*Flavobacterium psychrophilum*	DSM 21280	J.-F. Bernardet
12	*Klebsiella oxytoca*	DSM 5175	R. Hugh
13	*Klebsiella pneumoniae*	DSM 26371	H. Dalton
14	*Klebsiella pneumoniae*	DSM 30104	CDC, Atlanta; 298-53
15	*Listeria monocytogenes*	DSM 19094	H. Seeliger
16	*Mycobacterium phlei*	DSM 750	IPH
17	*Nocardia nova*	DSM 44481	N. F. Conant
18	*Proteus hauseri*	DSM 30118	K.B. Lehmann
19	*Proteus mirabilis*	DSM 4479	CDC PR 14
20	*Pseudomonas aeruginosa*	DSM 1128	C.P. Hegarty
21	*Pseudomonas aeruginosa*	DSM 1117	A. Madeiros
22	*Salmonella enterica*	DSM 19587	CDC
23	*Salmonella enterica*	DSM 17420	CDC
24	*Staphylococcus aureus*	DSM 2569	E.H. Gerlach
25	*Staphylococcus aureus*	DSM 1104	F. Schoenknecht
26	*Staphylococcus aureus*	DSM 46320	W. Witte
27	*Staphylococcus aureus*	DSM 799	AMC
28	*Streptococcus dysgalactiae*	DSM 20662	T.M. Higgs
29	*Staphylococcus epidermidis*	DSM 1798	FDA
30	*Streptococcus equi* ssp. *equi*	DSM 20561	R.E.O. Williams
31	*Staphylococcus felis*	DSM 7377	S. Igimi
32	*Staphylococcus intermedius*	DSM 20373	V. Hajek
33	*Streptococcus pyogenes*	DSM 11728	E. Mortimer
34	*Yersinia enterocolitica*	DSM 4780	J.M. Coffey

**Table 3:** Primers and probe used in the PCR for detection of *Devriesea agamarum*.

Forward Primer Devag16_For	GATGACTGCAGAGATGTGGTG
Reverse Primer Devag16_Rev	TTTGTACCGGCCATTGTAGCAT
Oligonucleotide probe FAM BHQ1	CATGTTGCCAGCACTTCGG

**Table 4:** Results of bacteriological culture and *Devriesea agamarum* PCR of clinical samples from 38 lizards.

Animal No.	Species	Clinical Signs and History and Additional Information	Country of Origin	Time from Sampling to Sample Preparation:	Sample Material	Bacterial Culture	PCR Result
1	*Uromastyx* sp.	Suspicious skin lesion Same wildlife park as animal 2	NL	unknown	Skin	N.D.	Positive (CT 23.71)
2	*Uromastyx* sp.	Suspected lesions/ dermatitis Same wildlife park as animal 1	NL	unknown	Swabs (skin)	N.D.	Positive (CT 30.53)
3	*Pogona* sp.	Black discoloration of the scales after shedding, especially on the tail.	NL	5d	Skin	+ *Glutamibacter* *creatinolyticus* + *Micrococcus* sp. + aerobic spore-forming bacteria	Negative
					Swab (skin)		Negative
4	*Uromastyx* sp.	Partner to animal 5	NL	2d	Swab (oral mucosa)	++ *Pantoea agglomerans* ++ *Pseudomonas* *aeruginosa* ++ aerobic spore-forming bacteria	Positive (CT 28.47)
5	*Uromastyx* sp.	Partner to animal 4	NL	2d	Swab (oral mucosa)	++ *Exiguobacterium* *mexicanum* ++ *Kluyvera intermedia* ++ *Pseudomonas* *chlororaphis* ++ aerobic spore-forming bacteria	Negative
6	*Uromastyx* sp.	Reported to have been treated with antibiotics	NL	2d	Swab (oral mucosa)	++ *Acinetobacter variabilis* +++ *Arthrobacter* *globiformis* + *Bacillus cereus*	Positive (CT 28.44)
7	*Pogona vitticeps*	No clinical signs	DE	1d	Swab (oral mucosa)	++ *Proteus mirabilis*	Positive (CT 33.17)
8	*Pogona vitticeps*	No clinical signs	DE	1d	Swab (oral mucosa)	+++ *Enterobacter cloacae* + *Pseudomonas* *aeruginosa*	Equivocal (CT 35.71)
9	*Pogona vitticeps*	No clinical signs Partner to animal 10 Confiscated	DE	1d	Swab (oral mucosa)	++ *Bordetella hinzii*	Negative
10	*Pogona vitticeps*	No clinical signs Partner to animal 9 Confiscated	DE	1d	Swab (oral mucosa)	+ *Bordetella hinzii* + *Peribacillus muralis* ++ *Enterococcus faecalis* + *Pantoea agglomerans*	Negative
11	*Pogona vitticeps*	No clinical signs Abandoned	DE	1d	Swab (oral mucosa)	++ *Morganella morganii* ++ *Klebsiella oxytoca*	Positive (CT 27.56)
12	*Pogona vitticeps*	No clinical signs Abandoned	DE	1d	Swab (oral mucosa)	++ *Klebsiella oxytoca* + *Proteus mirabilis*	Negative
13	*Pogona* *henrylawsoni*	No clinical signs Abandoned juvenile same enclosure as animals 14 and 15	DE	unknown	Swab (oral mucosa)	+ *Aeromonas hydrophila* (+) aerobic spore-forming bacteria	Negative
14	*Pogona* *henrylawsoni*	No clinical signs Abandoned juvenile same enclosure as animals 13 and 15	DE	unknown	Swab (oral mucosa)	++ *Proteus mirabilis* (+) aerobic spore-forming bacteria	Negative
15	*Pogona* *henrylawsoni*	No clinical signs Abandoned juvenile same enclosure as animals 13 and 14	DE	unknown	Swab (oral mucosa)	+ *Aeromonas hydrophila* (+) alpha-hemolytic streptococci	Negative
16	*Pogona vitticeps*	No clinical signs	DE	unknown	Swab (oral mucosa)	+ *Aeromonas hydrophila* + *Pseudomonas* *aeruginosa* (+) aerobic spore-forming bacteria	Positive (CT 27.20)
17	*Pogona vitticeps*	No clinical signs	DE	unknown	Swab (oral mucosa)	+++ *Klebsiella oxytoca*	Equivocal (CT 35.26)
18	*Pogona vitticeps*	No clinical signs	DE	unknown	Swab (oral mucosa)	++ *Klebsiella oxytoca*	Equivocal (CT 35.07)
19	Scincidae	Unknown	DE	2d	Skin	N.D.	Negative
20	Iguanidae	Hyperkeratosis (dorsal)	DE	2d	Skin	N.D.	Negative
21	*Uromastyx* sp.	Skin lesion	DE	2d	Skin	N.D.	Positive (CT 21.34)
22	*Uromastyx* sp.	Skin lesion Animals 22, 23 and 24 kept in the same enclosure	DE	unknown	Swab (outer skin of the mouth)	+ *Pseudomonas* *aeruginosa* + *Serratia marcescens*	Positive (CT 28.45)
					Swab (oral mucosa)	+ *Pseudomonas* *aeruginosa* + *Serratia marcescens*	Positive (CT 34.89)
					Skin	N.D.	Positive (CT 31.56)
					Swab (skin)	N.D.	Positive (CT 30.88)
23	*Uromastyx* sp.	Skin lesion Animals 22, 23 and 24 kept in the same enclosure	DE	unknown	Swab (outer skin of the mouth)	+ *Enterobacter cloacae*	Positive (CT 32.20)
					Swab (oral mucosa)	+ *Enterobacter cloacae*	Positive (CT 32.06)
					Skin	N.D.	Positive (CT 31.97)
					Swab (Skin)	N.D.	Positive (CT 34.05)
24	*Uromastyx* sp.	Skin lesion Animals 22, 23 and 24 kept in the same enclosure	DE	unknown	Swab (outer skin of the mouth)	+ *Pseudomonas* *synxantha*	Equivocal (CT 35.24)
					Swab (oral mucosa)	*+ Pantoea agglomerans*	Negative
					Skin	N.D.	Positive (CT 33.84)
					Swab (Skin)	N.D.	Equivocal (CT 35.75)
25	*Corucia zebrata*	Multiple animals with minimal to moderate stomatitis	NL	2d	Swab (skin)	N.D.	Negative
					Skin	+++ *Pseudomonas* *aeruginosa*	Negative
26	*Chlamydosaurus kingii*	No clinical signs Kept with animal 27	DE	5d	Swab (oral mucosa)	+ *Proteus mirabilis* *++ Serratia marcescens*	Negative
27	*Chlamydosaurus kingii*	No clinical signs Kept with animal 26	DE	5d	Swab (oral mucosa)	+ *Klebsiella oxytoca* + *Stenotrophomonas maltophilia* + aerobic spore-forming bacteria	Negative
28	*Laemanctus* *serratus*	No clinical signs Kept with animals 29 and 30	DE	5d	Swab (oral mucosa)	+ *Klebsiella oxytoca* + *Morganella morganii*	Positive (CT 31.20)
29	*Laemanctus* *serratus*	No clinical signs Kept with animals 28 and 30	DE	5d	Swab (oral mucosa)	+ *Deinococcus* *proteolyticus* + *Morganella morganii* + *Serratia marcescens*	Positive (CT 34.35)
30	*Laemanctus* *serratus*	No clinical signs Kept with animals 28 and 29	DE	5d	Swab (oral mucosa)	+ *Morganella morganii* + *Proteus mirabilis*	Positive (CT 29.10)
31	*Laudakia stellio picea*	No clinical signs Kept with animal 32	DE	5d	Swab (oral mucosa)	(+) aerobic spore-forming bacteria	Equivocal (CT 35.80)
32	*Laudakia stellio picea*	No clinical signs Kept with animal 31	DE	5d	Swab (oral mucosa)	+ *Pseudomonas* sp. + *Serratia marcescens* + aerobic spore-forming bacteria	Equivocal (CT 36.87)
33	*Pogona vitticeps*	No clinical signs Kept with animal 34	DE	5d	Swab (oral mucosa)	(+) *Staphylococcus* *epidermidis*	Positive (CT 34.07)
34	*Pogona vitticeps*	No clinical signs Kept with animal 33	DE	5d	Swab (oral mucosa)	+ *Staphylococcus aureus* (+) aerobic spore-forming bacteria	Positive (CT 32.61)
35	*Pogona vitticeps*	Juvenile No clinical signs Kept with animals 36, 37 and 38	DE	5d	Swab (oral mucosa)	(+) aerobic spore-forming bacteria	Negative
36	*Pogona vitticeps*	Juvenile No clinical signs Kept with animals 35, 37 and 38	DE	5d	Swab (oral mucosa)	+ *Achromobacter* *xylosoxidans*	Negative
37	*Pogona vitticeps*	Juvenile No clinical signs Kept with animals 35, 36 and 38	DE	5d	Swab (oral mucosa)	+ *Serratia marcescens* (+) aerobic spore-forming bacteria	Negative
38	*Pogona vitticeps*	Juvenile No clinical signs Kept with animals 35, 36 and 37	DE	5d	Swab (oral mucosa)	+ *Proteus mirabilis*	Negative

Legend: N.D. = not done, d = days, DE = Germany, NL = the Netherlands.

## Data Availability

Data is contained within the article.
